# Association of *CHRNA5-A3-B4* Variation with Esophageal Squamous Cell Carcinoma Risk and Smoking Behaviors in a Chinese Population

**DOI:** 10.1371/journal.pone.0067664

**Published:** 2013-07-02

**Authors:** Yang Wang, Haijian Wu, Qiji Liu, Cuihong Wang, Lei Fu, Han Wang, Wenjie Zhu, Weijiang Fu, Yajuan Lv, Shikun Wang, Likuan Hu

**Affiliations:** 1 Department of Radiation, Oncology Center, Qilu Hospital of Shandong University, Jinan, Shandong, People’s Republic of China; 2 Key Laboratory of Cardiovascular Remodeling and Function Research, Chinese Ministry of Education and Chinese Ministry of Public Health, Jinan, Shandong, People’s Republic of China; 3 Department of Medical Genetics School of Medicine, Shandong University, Jinan, Shandong, People’s Republic of China; 4 Department of Radiation, Shandong Tumor Hospital and Institute, Jinan, Shandong, People’s Republic of China; 5 Oncology Center, the Second Hospital of Shandong University, Jinan, Shandong, People’s Republic of China; 6 Oncology Department of Jinan Central Hospital, Shandong University, Jinan, Shandong, People’s Republic of China; The University of Texas MD Anderson Cancer Center, United States of America

## Abstract

**Background:**

*CHRNA5-A3-B4*, the gene cluster encoding nicotinic acetylcholine receptor subunits, is associated with lung cancer risk and smoking behaviors in people of European descent. Because cigarette smoking is also a major risk factor for esophageal squamous cell carcinoma (ESCC), we investigated the associations between variants in *CHRNA5-A3-B4* and ESCC risk, as well as smoking behaviors, in a Chinese population.

**Methods:**

A case-control study of 866 ESCC patients and 952 healthy controls was performed to study the association of polymorphisms (rs667282 and rs3743073) in *CHRNA5-A3-B4* with cancer risk using logistic regression models. The relationships between *CHRNA5-A3-B4* polymorphisms and smoking behaviors that can be quantified by cigarettes smoked per day (CPD) and pack-years of smoking were separately estimated with Kruskal-Wallis tests among all 840 smokers.

**Results:**

*CHRNA5-A3-B4* rs667282 TT/TG genotypes were associated with significantly increased risk of ESCC [adjusted odds ratio (OR) = 1.32, 95% confidence interval (CI) = 1.03 – 1.69, P = 0.029]. The increased ESCC risk was even higher among younger subjects (≤60 years) (OR = 1.44, 95% CI = 1.04 – 1.98, P = 0.024). These effects were not found in another polymorphism rs3743073. No evident association between the two polymorphisms and smoking behaviors was observed.

**Conclusions:**

These results support the hypothesis that *CHRNA5-A3-B4* is a susceptibility gene cluster for ESCC. The relationship between *CHRNA5-A3-B4* and smoking behaviors in a Chinese population needs further investigation.

## Introduction

Esophageal cancer is the eighth most common cancer in the world, and there were 482 300 newly diagnosed cases worldwide in 2008 [1], [2]. Of the two major histological types, esophageal squamous cell carcinoma (ESCC) rather than adenocarcinoma is common worldwide [3], and it is relatively more prevalent among the East Asian population, including the Chinese. Although the exact etiology of ESCC remains to be identified, cigarette smoking has been demonstrated as the major factor that contributes to the development of ESCC in accumulating epidemiological and clinical studies [1]. We can not exclude the possibility that genes in association with smoking may modify the susceptibility to ESCC.

The nicotinic acetylcholine receptor (nAChR) belongs to the superfamily of ligand-gated ion channels, and it is activated by acetylcholine (Ach), choline and nicotine. It is involved in the regulation of nicotine and nitrosamines [4]. Nicotine in tobacco triggers the psychological and neurobiological effects that are associated with smoking consumption and addiction [5], while nitrosamines are important carcinogens in tobacco that contribute to the development of many smoking-related cancers, including lung cancer and ESCC [6]. Furthermore, the nAChR pathway is related to tumor cell proliferation, apoptosis, survival, migration, invasion, and angiogenesis [7], [8], which can affect the progression of cancer patients. Currently, nAChR is thought to be an important regulator of a complex network of neurotransmitters that govern the synthesis and release of growth, angiogenic and neurotrophic factors in cancer cells, the cancer microenvironment and distinct organs [9].

Recently, genome-wide association (GWA) studies have shown three single nucleotide polymorphisms (SNPs) (rs1051730, rs16969968 and rs8034191 in the *CHRNA5-A3-B4* gene cluster, which encodes the nAChR subunits) to be related to lung cancer risk and smoking consumption and addiction behaviors in people of European descent [10]–[13]. Because these three variants are extremely rare in the Asian population, according to the HapMap database and a study of Wu et al [14], they play little roles, if any, in risk of smoking-related cancers and smoking behaviors in Chinese people. Interestingly, two independent studies found that variants rs667282 and rs3743073 of *CHRNA5-A3-B4* can affect lung cancer risk in a Chinese population [14], [15], similar to rs1051730, rs16969968 and rs8034191 in people of European descent. Consequently, we investigated the associations of the 2 SNPs at *CHRNA5-A3-B4* (rs667282 and rs3743073) with ESCC risk and smoking behaviors in a Chinese population, and we further explored the influence of *CHRNA5-A3-B4* polymorphisms on cancer progression.

## Materials and Methods

### Ethics Statement

The study is in compliance with the Helsinki Declaration, and was approved by the Ethics Committees of Qilu Hospital of Shandong University (approval number: KYLL2010058). At recruitment, all participants gave written informed consent.

### Study Sample

This study consisted of 866 ESCC patients and 952 healthy controls. All subjects were biologically unrelated ethnic Han Chinese from Shandong Province in China. Patients who were newly diagnosed with histologically confirmed primary ESCC were recruited from Qilu Hospital of Shandong University, Shandong Tumor Hospital and Institute and the Second Hospital of Shandong University from 2010 to 2012. Controls were healthy individuals selected from the physical examination center of Qilu Hospital during the same period and matched with the patients by age and gender.

### Clinical Data Collection

We collected subject data through face-to-face interviews conducted by trained medical students or clinical doctors with a pre-tested standardized questionnaire regarding age, gender, drinking status, smoking status, smoking dose, and smoking exposure. Smokers or drinkers were defined as those who had been regularly smoking or drinking for 1 year or longer in their lifetime, whereas nonsmokers or nondrinkers were defined as those who had not. ESCC patients were staged according to the American Joint Committee on Cancer (AJCC) tumor-node-metastasis (TNM) staging system whenever possible from operative specimens [16]. Because positron emission tomography and endoscopic ultrasonography were not required as a component of the pretreatment evaluation at the time of study, we staged inoperable patients, who were not applicable for the AJCC staging system, using computer tomography [17], [18]. Both staging methods are accepted by oncologists [19]–[21].

### Genotyping

Whole-blood samples were collected from all study participants. We extracted genomic DNA from the blood samples using the Tiangen Biotech kit (DP319; Tiangen Biotech (Beijing) Co., Ltd., Beijing China). Rs667282 and rs3743073 were genotyped by the 5' nuclease cleavage assay (TaqMan method) obtained from Applied Biosystems (assay ID: C__11942801_10 and C__25648144_10, respectively), which uses two allele-specific TaqMan MGB probes and a PCR primer pair to detect the specific SNP target. Following the manufacturer’s instructions, PCR amplifications were conducted in a 96-well Applied Biosystems 7500 Real Time PCR System, and allelic discrimination was performed using the SDS 1.4 software.

### Statistical Analysis

The genotype frequencies of each SNP were tested for deviation from the Hardy–Weinberg equilibrium using a goodness-of-fit chi-square test. This method was used to determine whether the samples were from a population and representative. The association of each SNP with ESCC risk was estimated using a logistic regression model with adjustment for age, sex, smoking status, and drinking status. Associations between the 2 SNP genotype groups with the histological grade and clinical stage of ESCC were evaluated using the chi-square test. The Kruskal–Wallis test was used to compare the difference of cigarettes per day (CPD) and pack-years of smoking. Individuals who denied ever smoking were excluded from this analysis, because they may have never had sufficient exposure to cigarette smoking to become addicted [11], [22]. Statistical analyses were performed using SPSS (version 20) and Stata (version 12.0). *P*<0.05 was the criterion of statistical significance, and all statistical tests were two-sided.

## Results

### Baseline Characteristics of the Study Population

The frequency distributions of the baseline characteristics and clinical features of the study population are presented in Table 1. There were no significant differences between cases and controls in terms of age or gender distribution (*P* = 0.86 and 0.75, respectively). In contrast, smoking and drinking were significantly more frequent in cancer patients compared to control subjects (both *P*<0.001). Among the ESCC patients, the fractions of histological grades from well to poor were 21.2%, 48.7% and 30.0%, respectively. Approximately 52.3% and 47.7% of ESCC patients were at the early clinical stage (I – II) and advanced stage (III – IV), respectively.

**Table 1:** The baseline characteristics of the study population.

Characteristic	ESCC subjects	Control subjects	*P* †
	n = 866	n = 952	
**Age, mean (SD)**	61.2 (8.8)	61.1 (9.0)	0.86
**Gender, N (%)**			
Male	746 (86)	825 (87)	0.75
Female	120 (14)	127 (13)	
**Smoking status, N (%)**			
Never smoker	322 (37)	656 (69)	<0.001
Current smokers	416 (48)	243 (25.5)	
Former smokers	128 (15)	53 (5.5)	
**Drinking status, N (%)**			
Never drinking	333 (38)	597 (63)	<0.001
Ever drinking	533 (62)	355 (37)	
**Smoking variables,** **mean (SD)**			
Cigarettes per day	23.1 (11.4)	22.2 (11.5)	0.22
Smoking duration in years	32.5 (9.7)	31.5 (9.5)	0.13
Pack–years	37.0 (20.2)	33.9 (19.8)	0.01
**Differentiation, N (%)**			
Well	184 (21.2)		
Moderate	422 (48.7)		
Poor	260 (30.0)		
**Clinical stage, N (%)**			
I – II	453 (52.3)		
III – IV	413 (47.7)		

† Two-sided χ2 test for the categorical variables (sex, smoker status and drinking status), and Kruskal-Wallis test for the continuous variables (age and smoking variables).

### Association between *CHRNA5-A3-B4* Polymorphisms and ESCC Risk

The genotype distribution of rs667282 and rs3743073 among cases and controls and their association with ESCC risk are shown in Table 2. The observed genotype frequencies for both polymorphisms were in Hardy-Weinberg equilibrium among the controls (*P* = 0.33 for rs667282 and *P* = 0.18 for rs3743073). The genotypes of rs667282 were markedly distinct in cases and controls irrespective of age, gender, smoking status and drinking status, whereas the allele distribution of rs3743073 was similar between cases and controls. We calculated the odds ratios adjusted by age, sex, drinking status and smoking status. The TT/TC genotypes of rs667282 exhibited an increased association with ESCC (adjusted OR = 1.32, 95%CI = 1.03 to 1.69, *P* = 0.029). Rs3743073 did not show a significant association with the risk of ESCC.

**Table 2:** The genotype distribution of 2 SNPs among cases and controls and their association with ESCC risk.

Genotype group	ESCC subjects	Control subjects	Adjusted OR (95% CI)†	*P*
	n = 866	n = 952		
**rs667282, N (%)**				
CC	156 (18.0)	205 (21.6)	Reference	
TC	419 (48.4)	457 (48.1)	1.29 (0.99–1.67)	0.062
TT	291 (33.6)	289 (30.4)	1.36 (1.03–1.80)	0.030
TT/TC	710 (82.0)	746 (78.5)	1.32 (1.03–1.69)	0.029
**rs3743073, N (%)**				
TT	225 (26.1)	271 (28.6)	Reference	
TG	424 (49.2)	453 (47.7)	1.15 (0.91–1.46)	0.249
GG	213 (24.7)	225 (23.7)	1.10 (0.84–1.45)	0.470
GG/TG	637 (73.9)	678 (71.4)	1.13 (0.91–1.41)	0.267

† adjusted by age, gender, drinking status and smoking statusin logistic regression model. CI: confidence interval; OR: odds ratio.

We further examined the impact of *CHRNA5-A3-B4* (rs667282) on ESCC risk, stratified by age, gender, drinking status and smoking status (Table 3). The increased cancer risk accompanied by rs667282 TT/TC genotypes was more notable in younger subjects (≤60 years) (OR = 1.44, 95%CI = 1.04 to 1.98, *P* = 0.024). No significant discrepancy was observed in the stratification of gender, drinking status and smoking status.

**Table 3:** Stratification analysis of rs667282 genotypes by selected variables in ESCC patients and controls.

Category	ESCC subjects		Control subjects		OR (95% CI) *	*P*
	CC	TT/TC	CC	TT/TC		
**Age**						
≤60	80	358	118	366	1.44 (1.04–1.98)	0.024
>60	76	352	88	379	1.07 (0.77–1.51)	0.675
**Sex**						
Male	139	607	177	647	1.19 (0.93–1.53)	0.160
Female	17	103	29	98	1.79 (0.93–3.47)	0.083
**Smoking**						
Never smoker	56	266	136	520	1.24 (0.88–1.75)	0.217
Lighter smoker †	72	298	53	154	1.42 (0.95–2.13)	0.087
Heavier smoker ‡	28	146	17	71	1.25 (0.64–2.43)	0.514
**Drinking**						
Never drinker	58	275	122	475	1.21 (0.86–1.72)	0.264
Drinker	98	435	84	270	1.38 (0.99–1.92)	0.054

† the smoker with CPD≤20.

‡ the smoker with CPD>20.

* CI: confidence interval; OR: odds ratio.

### Association of *CHRNA5-A3-B4* Polymorphisms with Histological Grade and Clinical Stage of ESCC Patients

Because the *CHRNA5-A3-B4* gene cluster has been suggested to influence cancer progression, we also investigated the association of the 2 SNPs in *CHRNA5-A3-B4* with the histological grade and clinical stage of ESCC patients. As shown in Table 4, we found that there was a weak association between the TT/TC genotypes of rs667282 and advanced clinical stage (OR = 1.30, 95%CI = 0.78 to 1.85, *P* = 0.137). No association between *CHRNA5-A3-B4* polymorphisms and the histological grade of ESCC was observed in this study.

**Table 4:** Association between 2 SNPs genotype groups with histological grade and clinical stage of ESCC.

Category	rs667282				rs3743073			
	CC	TT/TC	OR (95% CI)*	*P*	TT	GG/TG	OR (95% CI) *	*P*
**Differentiation, N (%)**								
Well	35	149	Reference		46	138	Reference	
Moderate	76	346	1.06 (0.68–1.67)	0.767	115	306	0.89 (0.60–1.32)	0.553
Poor	45	215	1.12 (0.69–1.83)	0.643	64	193	1.01 (0.65–1.56)	0.981
**Clinical stage, N (%)**								
I – II	90	363	Reference		122	330	Reference	
III – IV	66	347	1.30 (0.78–1.85)	0.137	103	307	1.10 (0.81–1.50)	0.533

* CI: confidence interval; OR: odds ratio.

### Association between *CHRNA5-A3-B4* Polymorphisms and Smoking Behaviors

We analyzed the association of *CHRNA5-A3-B4* polymorphisms with smoking behaviors for 840 smokers among all participants. The values of CPD and pack-years were used as the measurements of smoking behaviors for this analysis because they are frequently used in GWA studies [11]–[13]. Table 5 shows the results for smoking behaviors. Subjects with the *CHRNA5-A3-B4* (rs667282) TT/TC genotypes exhibited a tendency to smoke more CPD than those with the CC genotype (mean = 23.1 CPD, 95%CI = 22.3 to 24.0 CPD; mean = 21.5 CPD, 95% CI = 19.9 to 23.2 CPD, respectively), and more pack-years (mean = 36.3 pack-years, 95%CI = 34.7 to 47.8 pack-years; mean = 34.6 pack-years, 95%CI = 31.6 to 37.6 pack-years, respectively). However, this difference did not reach statistical significance (*P* = 0.135 and 0.233, respectively). Genotypes of rs3743073 were not associated with CPD and pack-years of smoking.

**Table 5:** Association between 2 SNPs genotype groups with cigarettes per day (CPD) and pack-years in all smoking participants.

Genotype group	N	CPD (95% CI)	*P* †	Pack-years (95% CI)	*P* †
rs667282 TT/TC	668	23.1 (22.3–24.0)	0.135	36.3 (34.7–47.8)	0.233
rs667282 CC	170	21.5 (19.9–23.2)		34.6 (31.6–37.6)	
rs3743073 GG/TG	609	23.1 (22.2–24.0)	0.304	36.0 (34.4–37.5)	0.430
rs3743073 TT	227	22.0 (20.6–23.5)		35.7 (32.9–38.4)	

† *P* values were calculated by the Kruskal-Wallis test and were two-sided.

## Discussion

In this hospital-based case-control study of Chinese individuals, we found that the SNP rs667282 in *CHRNA5-A3-B4*, the gene cluster encoding nAChR subunits (alpha3, alpha5, and beta4), exhibited significant associations with ESCC risk. These nAChR subunits are the principle targets of nicotine, and can also bind to two nicotine-specific metabolites namely: 4-(Methylnitrosamino)-1- (3-pyridyl)-1- butanone (NNK) and N-nitrosonornicotine (NNN) [12], [23], which are potent carcinogens and the effects of which on DNA are regarded as the primary cause of smoking-related cancers [24]. As the agonists for nAChRs, NNK and NNN have been found to induce many types of smoking-related cancers in laboratory animals [25], [26]. Recently, Yuan et al. found that NNN exhibited a significant and important role in esophageal carcinogenesis in humans [27]. Furthermore, NNN showed a 5 000× higher affinity for nAChRs compared to nicotine [28]. Thus, it is biologically plausible that variants in the gene cluster confer individuals’ susceptibility to esophageal cancer, especially ESCC.

The relationship between cancer and carcinogen-related genes has been well established in many types of cancers [29]–[31]. Recently, a GWA study in a Japanese population showed that two SNPs in the *ALDH2* and *ADH1B*, which encode dehydrogenases involved in alcohol metabolism, were strongly associated with ESCC risk [32]. These results indicate that ESCC may also be influenced by genetic polymorphisms of carcinogen-related genes, although *ALDH2* and *ADH1B* did not exhibit associations with ESCC in a subsequent study of Chinese subjects [33].

In the stratified analysis, associations between rs667282 genotypes and ESCC risk were stronger for younger subjects, lighter smokers or drinkers. One thing to note is the higher risk tendency for lighter smokers than heavier smokers. The pattern of higher genetic risk in the low-carcinogen exposure stratum supports the hypothesis that genotype contributes to the risk of carcinogenesis directly, rather than simply indirectly through an association with altered smoking quantity. This phenomenon has also been observed for the key SNP rs1051730 in *CHRNA5-A3-B4* in lung cancers in people of European descent [34], [35]. In addition, we also found that rs667282 tended to be a risk factor for advanced tumor clinical stage, although the influence was not significant. To date, studies investigating the *CHRNA5-A3-B4* polymorphisms in relation to tumor clinical stage have been few; however, it has been proposed that nicotine after initiation may contribute to the progression phase of cancer development because nicotine promotes the growth of cancer cells and the proliferation of endothelial cells in vivo [36]. Furthermore, a negative correlation between *CHRNA5-A3-B4* polymorphisms and lung cancer survival has been shown in many studies [37], [38]. If the tendency can be confirmed by additional studies with a larger sample, rs667282 might help to accurately predict the clinical course of ESCC.

In people of European descent, variants in the *CHRNA5-A3-B4* cluster showed a strong association with smoking consumption. Recently, in a functional magnetic resonance study of smokers, a genetic variation in *CHRNA5* was observed to affect reactivity to images of smoking in brain areas related to memory and habitual behaviors, such as the hippocampus and dorsal striatum [39]. However, the relationship between *CHRNA5-A3-B4* and smoking consumption was not significant in this study, which was consistent with the study by Niu et al. regarding strong lung cancer-related variants in *CHRNA5-A3-B4* and smoking behaviors in Chinese people [15]. Wu et al. reported nominally significant associations between rs667282 and CPD in a study of 3 725 smokers in a Chinese population (*P* = 0.022) [14]. In a study using an even larger sample (8 842 smokers) in Korea, the association between variants in the *CHRNA5-A3-B4* and smoking quantity was also weak (*P* = 0.008–0.028) [40]. It seems that this smoking association is much weaker in an Asian population. Reasons about the racial difference in the gene cluster with smoking associations are unclear. Differences in genetic and environmental backgrounds may contribute to this discrepancy.

Several limitations of this study should be mentioned. First, some data on cigarette consumption were retrospective, so there may be some recall bias. Moreover, because of the limited sample number, the effect of the variants on ESCC risk in the stratified analysis did not reach statistical significance. A large population-based study is still needed to confirm the conclusions in this study.

In summary, to the best of our knowledge, this study was the first to demonstrate that the *CHRNA5-A3-B4* gene cluster was associated with increased ESCC risk. This finding reveals another case study of a gene-environment interaction, highlighting the role of carcinogen-related genes in the pathology of ESCC. Further studies are required to confirm this finding in different ethnic populations.
